# MGV-seq: a sensitive and culture-independent method for detecting microbial genetic variation

**DOI:** 10.3389/fmicb.2025.1603255

**Published:** 2025-06-25

**Authors:** Lun Li, Weiyu Kong, Jing Sun, Yongzhong Jiang, Tiantian Li, Zhihui Xia, Junfei Zhou, Zhiwei Fang, Lihong Chen, Shun Feng, Huiyin Song, Huafeng Xiao, Baolong Zhang, Bin Fang, Hai Peng, Lifen Gao

**Affiliations:** ^1^Institute for Systems Biology, Jianghan University, Wuhan, Hubei, China; ^2^Hubei Provincial Center for Disease Control and Prevention, Wuhan, Hubei, China; ^3^Hainan Key Laboratory for Sustainable Utilization of Tropical Bioresources, College of Tropical Crops, Hainan University, Haikou, Hainan, China; ^4^Mingliao Biotechnology Co., Ltd., Wuhan, Hubei, China; ^5^Wuhan Zhongwei Gene Technology Co., Ltd., Wuhan, Hubei, China

**Keywords:** microbial genetic variation, multiple dispersed nucleotide polymorphism markers, multiplex polymerase chain reaction, MGV-Seq, strain purification, identification authentication

## Abstract

**Background:**

Precise detection of microbial genetic variation (MGV) at the strain level is essential for reliable disease diagnosis, pathogen surveillance, and reproducible research. Current methods, however, are constrained by limited sensitivity, specificity, and dependence on culturing. To address these challenges, we developed MGV-Seq, an innovative culture-independent approach that integrates multiplex PCR, high-throughput sequencing, and bioinformatics to analyze multiple dispersed nucleotide polymorphism (MNP) markers, enabling high-resolution strain differentiation.

**Methods:**

Using *Xanthomonas oryzae* as a model organism, we designed 213 MNP markers derived from 458 genome assemblies. Method validation encompassed reproducibility, accuracy, sensitivity (detection limit), and specificity using laboratory-adapted strains, artificial DNA mixtures, and uncultured rice leaf samples. Performance was benchmarked against whole-genome sequencing (WGS) and LoFreq variant calling.

**Results:**

MGV-Seq achieved 100% reproducibility and accuracy in major allele detection, with sensitivity down to 0.1% (*n* = 12 strains) for low-abundance variants and significantly higher specificity than LoFreq. Analysis to 40 *X. oryzae* strains revealed widespread heterogeneity (90% of strains) and misidentification (e.g., HN-P5 as *Xoc*). Homonymous strains exhibited significant genetic and phenotypic divergence, attributed to contamination rather than mutation. MGV-Seq successfully identified dominant strains and low-frequency variants in rice leaf samples and authenticated single-colony strains with 100% major allele similarity.

**Conclusion:**

MGV-Seq establishes a robust, high-throughput solution for strain identification, microevolution monitoring, and authentication, overcoming limitations of culture-dependent and metagenomics-based methods. Its applicability extends to other microorganisms, offering potential for clinical, agricultural, and forensic diagnostics.

## 1 Introduction

Plant pathogens severely damage crops, vegetables, and fruits. Based on the infected hosts and pathogenic traits, plant pathogenic species can be classified into subspecies, pathovar, subpathovar, and strains, representing considerable variability in their pathogenicity (Rademaker et al., 2005). Therefore, accurate and sensitive detection of taxonomic variations and accurate identification of phytopathogenic microorganisms at the strain level in unprocessed host samples are crucial for microbe-related disease diagnosis and prevention. However, the genetic and phenotypic similarity of taxa beyond species level challenges isolation and culturing, limiting the effectiveness of conventional biochemical, pathogenicity and serological analyses (Catara et al., 2021).

Identification of microbial strains is also essential for plant research involving phytopathogenic microorganisms. A well-curated strain is generally regarded as a genetically homogeneous population. However, laboratory-adapted microbial strains are usually used for laboratory-based research for a long time or distributed to laboratories worldwide. All the issues associated with cell lines—spontaneous mutation, contamination, and mislabeling—are likely to appear in phytopathogenic microorganisms during culture, storage, propagation, and passage (Andreu and Gibert, 2008, Feng et al., 2023, Zeng et al., 2023). The heterogeneity of cell lines has led to incomparable, inconsistent, or even contradictory results (Huang et al., 2017, Liu et al., 2019, Lorsch et al., 2014, Masters, 2012, Zeng et al., 2023). The importance of cell line authentication has attracted a great deal of attention lately. However, these critical issues have rarely been considered in studies and applications using phytopathogenic microorganisms. Technically, addressing this problem is also a challenge to existing approaches.

The existing approaches for detecting genetic variations within and across microorganisms can be divided into marker-dependent and marker-free categories. Marker-dependent approaches differentiate strains based on their genetic marker genotypes. Based on how markers are genotyped, they can be further categorized by length-based methods, such as multilocus variable-number tandem repeat analysis and pulsed-field gel electrophoresis analysis (Grissa et al., 2008, Machado et al., 2014, Poulin et al., 2015), and sequence-based methods, including multilocus sequence typing (MLST), DNA barcoding method such as 16S rRNA gene (16S) and internal transcribed spacer (ITS) (Antil et al., 2023), as well as whole-genome sequencing-single-nucleotide polymorphism (WGS-SNP) (Machado et al., 2014, Maiden, 2006, Roychowdhury et al., 2019, Saltykova et al., 2018). Length-based methods require minimal equipment and are convenient to use. However, they can only identify obvious differences in the length of the tested markers; they fail to detect nucleotide modifications and are dependent on the simultaneous experimentation of standard samples.

In contrast, the sequence-based MLST and DNA barcoding methods both utilize PCR amplification followed by targeted sequencing to examine nucleotide variations in a limited set of genetic markers. The MLST method focuses on a small number of species-specific markers (typically <10 loci) in several evolutionarily conserved housekeeping genes (Hajri et al., 2012, Maiden, 2006), and thus is only suitable for long-term monitoring and cannot track variations that occur over a short period. DNA barcoding method targets one or two fast-evolving regions within conserved genes (e.g., 16S, ITS), and are widely employed for cross-species identification. Nevertheless, this method is limited in distinguishing closely related taxa or strains due to insufficient sequence divergence. Furthermore, its reliance on minimal genetic markers makes results susceptible to PCR amplification failures caused by primer-template mismatches.

On the otherhand, the WGS-SNP technology can detect thousands of SNPs across genomes of microbes (Rademaker et al., 2005). However, SNPs are typically biallelic markers; therefore, they are inadequate to represent the underlying allelic diversity of a microbial population. Furthermore, SNP genotyping error rates are estimated to be as high as 0.9–7.9% (Bayer et al., 2017, Fang et al., 2017, Li, 2016, Sasaki et al., 2018), making it difficult to accurately detect variations in frequencies lower than the technical error rates. Consequently, WGS-SNP technology remains largely restricted to applications involving homogeneous, isolated strains. However, only 0.1–1% of microorganisms have been reported to be cultivated (Solden et al., 2016). Coupled with the bottleneck of culture isolation, culture-based approaches often result in biased selection of the dominant pathogenic species or genotype. In addition, they are time-consuming, ineffective, and unreliable for identifying strains or detecting sample variations.

In contrast, metagenomic next-generation sequencing (mNGS) is not limited to molecular markers across genomes and is usually applied to characterize uncultured microbial communities. However, mNGS requires ultra-large data sequencing, anywhere from 7.98 to 18.00 Gbp (Newberry et al., 2020), and intensive data computing to accurately distinguish the true targets from the numerous common epiphytic and entophytic bacterial species associated with plants. Furthermore, the background bacteria are often found in pathogen testing laboratories (Newberry et al., 2020), resulting in limited resolution at the species level and a limited capability to detect variations in taxon units beyond species (Jongman et al., 2020).

For accurate, efficient, and sensitive detection of microbial genetic variation (MGV), we developed MGV-Seq, a culture-independent MGV detection method. MGV-Seq combines multiplex polymerase chain reaction (PCR) amplification profiling, high-throughput sequencing, and a customized computational pipeline to capture all variations in hundreds of multiple dispersed nucleotide polymorphism (MNP) markers. MNP markers are a novel type of DNA marker consisting of multiple SNPs dispersed within short genomic segments, i.e., 250 nucleotides (Fang et al., 2021, Gao et al., 2023). Crucially, since this length falls within the sequencing read length, the haplotypes (i.e., the specific combination of multiple SNPs) can be directly derived from individual sequencing reads. Theoretically, the diversity of haplotypes increases exponentially with the number of SNPs covered, providing greater higher discriminative power for capturing diverse alleles within complex microbial communities. Coupled with multiplex PCR amplification of hundreds of genome-wide MNP markers and high-throughput sequencing, MGV-Seq is capable of simultaneous acquisition of high-resolution genetic profiles for the target microbe in an uncultured sample, avoiding time-consuming culture isolation processes. Furthermore, our integration of a statistical model to accurately capture alleles across varying abundance levels from sequencing data offers an ideal approach for strain identification, strain authentication, and monitoring strain microevolution.

In this study, we used the top bacterial plant pathogen *Xanthomonas oryzae* (Mansfield et al., 2012), which contains two rice pathovars, *oryzae* (*Xoo*) and *oryzicola* (*Xoc*), as a model microorganism (Niño-Liu et al., 2006). We first proved the high reproducibility and accuracy of MGV-Seq for identifying pathogens at the strain level in well-curated *X. oryzae* strains, as well as its high sensitivity and specificity for detecting low-abundance variations in artificial mixtures of strain DNA. We then applied MGV-Seq to detect the widespread genetic variations within laboratory strains and the microevolution between homonymous strains from the same parent, after which we sensitively identified *X. oryzae* strains and low-abundance variations directly from uncultured rice leaf samples. Moreover, we demonstrated the feasibility of MGV-Seq-based strain authentication to ensure the genetic similarity between daughter lines and the standard strain. Although MGV-Seq was used to analyze one phytopathogenic microorganism, we propose its broad applicability to other microorganisms.

## 2 Materials and methods

### 2.1 DNA extraction of *X. oryzae* strains

The P1–P10 strains from the International Rice Research Institute (IRRI) and the daughter lines from the Beijing, Wuhan, and Hainan laboratories used in this study are referred to as IRRI-P1–IRRI-P10, BJ-P1–BJ-P10, WH-P1–WH-P10, and HN-P1–HN-P10, respectively (Supplementary Figure S1). DNA of the IRRI-P1 to IRRI-P10 strains was provided by the IRRI laboratory. For DNA preparation of strains BJ-P1 to BJ-P10, WH-P1 to WH-P10, and HN-P1 to HN-P10, the cryopreserved bacterial cultures were reactivated via streaking on Potato-Sugar-Agar (PSA) medium without single-colony purification in their respective storage laboratories. After 72 h of incubation at 28°C, 100 mL of the activated inoculum was collected for DNA extraction using Rapid Bacterial Genomic DNA Isolation Kit (B518225, Sangon Biotech, China). For DNA preparation of rice leaf samples, rice leaves exhibiting bacterial blight symptoms were collected from the same paddy field area. Approximately 3 cm of tissue from the junction between diseased and healthy regions was excised. Ten leaves were pooled, and their surfaces were first cleaned with sterile water. Subsequently, DNA was extracted from the tissue powder using the aforementioned rapid bacterial genomic DNA extraction kit. DNA quantity was measured using Qubit Fluorometric Quantitation (Thermo Fisher Scientific, MA, United States).

### 2.2 Virulence evaluation of Xoo strains

The cryopreserved *Xoo* strains were reactivated by inoculation on Potato-Sucrose-Agar (PSA) medium and cultured at 28°C for 72 h. The activated bacterial cells were suspended in sterile water to prepare a bacterial suspension with a concentration of approximately 10^9^ CFU/mL. At the rice tillering stage, inoculation was performed using the leaf-clipping method on 4–5 fully expanded leaves per plant. Lesion length (LL) was measured on 10–15 infected leaves from five biological replicates 12 days post-inoculation. LL ≥10 cm, 10>LL ≥5 cm, and LL <5 cm were classified as susceptible, moderately resistant, and resistant, respectively, based on the disease rating system for LL (Quibod et al., 2020). We performed two-tailed Student’s *t*-tests to compare lesion lengths between experimental groups, applying a stringent significance threshold (*p* < 0.005) with Bonferroni correction for multiple comparisons when applicable.

### 2.3 Design of MNP markers and multiplex PCR primers

The design of the MNP markers and multiplex PCR primers for *Xanthomonas oryzae* followed our previous report with minor adjustments (Fang et al., 2021, Gao et al., 2023). The P6 (PXO99A) genome (Salzberg et al., 2008) was corrected using the genome data of WH-P6 and used as the synthetic reference genome to call SNPs in 458 *X. oryzae* strains using Bowtie2 (version 2.1.0) (Langmead and Salzberg, 2012). A sliding window of 275 base pairs (bp) was used for genome scanning with a 5 bp increment for the MNP loci design. The windows with discriminative power, which were defined as *t/c(N,2)*, where *c(N,2)* is the number of pairs among *N* strains used, and *t* is the number of pairs, each of which had at least two dispersed SNPs within the window, ≥0.1, were chosen for the multiplex PCR primer design. Primers were designed using Ion AmpliSeq Designer^1^ and synthesized by Thermo Fisher Scientific (Waltham, MA, United States).

### 2.4 Library construction and high-throughput sequencing

For all the samples in this study (including *Xoo* strains and rice leaf samples), sequencing libraries were constructed from 10 ng genomic DNA per sample using the Ion AmpliSeq Library Kit 2 (Cat# 4475345, Thermo Fisher Scientific, United States). The constructed amplicon library was quantified using the TaqMan probe method and mixed in equimolar amounts for sequencing on an Ion S5 sequencer (A27212, Thermo Fisher Scientific, Waltham, MA, United States) by single-end sequencing with a 300-bp read length.

### 2.5 Estimation of the reproducibility and accuracy of MGV-seq

Three technical repeats and two sequencing repeats of 12 strains (WH-P1, BJ-P1, HN-P1, WH-P2, BJ-P2, HN-P2, WH-P9, BJ-P9, HN-P9, WH-P10, BJ-P10, and HN-P10) were performed to estimate the accuracy and reproducibility of MGV-Seq. At the first sequencing batch, each of the 12 strains was prepared in three biological replicates (designated as rep-1, rep-2, and rep-3), thus a total of 12×3 = 36 technical repeats of 12 strains were generated. At the second sequencing batch, an additional replicate for the same 12 strains was performed (designated as rep-4). Consequently, this design yielded 3 batch replicates per strain (rep-1 vs. rep-4, rep-2 vs. rep-4, rep-3 vs. rep-4), resulting in a total of 36 batch replicates across all 12 strains (12 strains × 3 comparisons each). If the major allele of one MNP locus between two repeats is identical, the MNP locus is reproducible. The reproducibility of MGV-Seq was calculated as the ratio of the number of reproducible pairs to all genotype pairs. The reproducible major alleles were considered correct because the chance that the major alleles of one MNP locus detected by two repeats were consistent but incorrect should be relatively small. Therefore, the accuracy of MGV-Seq for detecting major alleles was 0.5+0.5* reproducibility.

### 2.6 A containerized computational tool for MGV-Seq

The tool containerized the computation via the following steps:

#### 2.6.1 MNP allele typing

FASTX-Toolkit (version 0.0.14)^2^ was used to pre-process the raw reads. First, base pairs with low-quality scores (<20) were trimmed, and the resultant reads <50 bp were removed. Then, the processed reads were mapped to a synthetic reference (see Design of MNP marker and multiplex PCR primer subsection) using Bowtie2 (version 2.1.0).

For each read that covered the entire genomic region of an MNP locus, the sequence located within the locus was used as its allele. To reduce errors caused by incorrect read alignment, any variation within consecutive mismatches with reference (mismatch interval ≤2 bp), repeat sequences (SSR), indels, and their flanking 2 bp of an allele were ignored. In addition, loci with at least one typed read were considered detected loci, and those with >100 typed reads were deemed valid MNP loci.

#### 2.6.2 MNP genotyping

Valid MNP loci were genotyped (Supplementary Figure S2). Briefly, all alleles identified in a valid MNP locus were referred to as candidate alleles of the locus in a microorganism strain. The candidate allele with the most supported reads was the major allele of the MNP locus in the strain if it was supported by at least 50% of the mapped reads on the locus; otherwise, this locus was excluded from the following analysis.

The remaining candidate alleles were a mixture of genuine minor alleles at lower frequencies and artifacts caused by PCR or sequencing errors. To distinguish genuine alleles from false ones, candidate alleles were first subjected to strand bias filters: (1) if the strand bias was greater than 10-fold or the differences between the strand bias of the candidate allele or that of the major allele were more than five-fold, the candidate allele was removed; (2) Fisher’s exact tests were applied to determine the strand bias, and candidate alleles with multiple tests corrected *p*-values <0.05 were abandoned.

Subsequently, a statistical model was applied. A candidate allele with *c* supporting reads was primarily assumed to be the product of the major allele solely due to amplicon sequencing errors. Under this hypothesis, the number of supporting reads of a candidate allele follows a binomial distribution with parameters *d* (marker sequencing depth) and *e* (amplicon sequencing error rate). If the candidate allele had an excessively larger number of supporting reads than expected, namely, if P(*X*≥*c*) was significantly low, the null hypothesis was overridden. When multiple candidate alleles were tested, the *p*-values were corrected for multiple testing, and candidate alleles with an FDR <0.5% were deemed true minor alleles.

#### 2.6.3 Estimation of genotyping parameters of MGV-Seq

Amplicon sequencing errors supposedly occur independently across reads; thus, the probability of multiple errors occurring is lower within a read sequence than that of a single error. Therefore, the parameter *e* used in the statistical model depends on the number of SNPs (*n*) between the candidate allele and major allele. The minor alleles detected at the homozygous MNP loci can be regarded as products of technical error. In this study, the parameter *e* for each *n*, denoted as *e* (*n*), had the highest ratio of reads that an erroneous allele bearing *n* SNPs could take up at a homogeneous MNP locus. Microorganisms typically live in populations, and monoclonal organisms are often composed of individuals of multiple generations. Therefore, the homozygous MNP loci in microorganisms could not be validated. Note that rice is a diploid plant, and F_1_ hybrids are produced by hybridization. The MNP loci identified as homozygous in both parents of hybrids are expected to be homozygous in the hybrid. Any minor alleles identified at these MNP loci can be attributed to amplicon sequencing errors. Therefore, we utilized the minor alleles detected at the expected homozygous MNP loci in the individual leaves of rice plants by amplicon sequencing to estimate the parameters [*e* (*n*1)] and MNPs [*e* (*n*1)]. To minimize sampling errors, only loci with depth >1,000 were considered. Based on the 930 MNP markers of the 16 individual rice plants, *e* (*n* = 1) and *e* (*n* > 1) were estimated to be 1.03 and 0.0994%, respectively.

### 2.7 Minor alleles detected by LoFreq

As reported previously (Wilm et al., 2012), a two-step strategy was used to call minor alleles using LoFreq. We first obtained consensus sequences from the original mapping results and used them as a reference in the second mapping round. Then, the final mapping results were analyzed using LoFreq (version 2.1.4).

### 2.8 Strain identification and authentication

The MNP genotypes of a tested strain or sample were compared with those of the strain to be compared using in-house scripts to obtain the major allele similarity (MAS) between the two, i.e., the ratio of the number of MNP loci with the same major alleles to the number of MNP loci detected commonly. For strain identification, the MNP genotypes of the tested samples were compared with those of the conspecific strains. The conspecific strain in the comparison group with the highest MAS was identified as the dominant strain in the tested sample. For strain authentication, the MNP genotypes of the tested strains were compared with those of the standard strains. The MAS threshold for strain authentication was established based on experimental requirements. In this study, we used a very stringent criterion, i.e., we considered that the strains were adequately authenticated when they had 100% MAS.

### 2.9 WGS

Whole genomes of IRRI-P1–IRRI-P10 were sequenced at Molbreeding Biotechnology Co., Ltd (Shijiazhuang, Hebei, China) on the Illumina HiSeq X Ten platform (Illumina, Inc., San Diego, CA, United States) with 150 bp read length. In addition, DNA libraries were constructed using the DNA library kit V3.1 (Molbreeding Biotechnology Co., Ltd.).

### 2.10 Sanger sequencing validation of MNP genotypes

Genotypes of the MNP loci among samples were validated using the same primers used in MGV-Seq to amplify the MNP loci, and Sanger sequencing was used for the amplification products (TsingKe Company, Wuhan, Hubei, China).

### 2.11 WGS alleles and published datasets

The WGS reads were first mapped to the reference genome using Bowtie2 (version 2.1.0) (Langmead and Salzberg, 2012) and consensus sequences within the MNP loci were considered major alleles. Nucleotides with <20 reads were masked to mitigate errors caused by low sequencing depths. Furthermore, the reference sequence of the MNP loci was compared with published assemblies using BLAST (version 2.2.26), and the most similar sequences were taken as the major alleles. Finally, the minor alleles detected using WGS were called by LoFreq using the downloaded complete genomes as respective references (see “Minor alleles detected by LoFreq” subsection).

### 2.12 Statistical analysis

All statistical analyses were conducted in R (version 3.5.1). Student’s *t*-tests and Fisher’s exact tests were performed by “t.test” function and “fisher.test” functions, respectively. Cluster analysis based on MNP genotypes was carried out as follows. First, pairwise genetic distances between strains were calculated as the complement of MAS (defined in section 2.8). On the basis of the calculated distance matrix, hierarchical clustering was then carried out using R’s “hclust” function. Finally, the resulting cluster dendrogram was visualized using the “ggtree” package (version 1.12.7).

## 3 Results

### 3.1 Design and characterization of MGV-Seq

#### 3.1.1 MGV-Seq—the method

We designed the MGV-Seq method to profile MGVs. The MGV-Seq procedure began with the selection of MNP markers (Fang et al., 2021) using the (re)sequencing data of representative strains from a species (see Materials and methods section). Briefly, the genome database of target species was constructed by collecting genome assemblies of conspecific strains from a public database, such as National Center for Biotechnology Information. Then, the genome assembly of a representative strain was used as the reference genome to call SNPs in the other genome assemblies, and a sliding window was used to scan those genomes assemblies to screen windows with at least two SNPs. The length of the sliding window and step between windows is determined based on the length of the amplicon that can be processed by the sequencer used. The windows with high discriminative power within species and uniform distribution in the reference genome were selected as candidates of MNP markers (see Materials and methods section). Primers for the multiplex amplification of the candidate MNP markers were designed using the available tools and synthesized after adding adaptors adapted to the sequencer used. Using the synthesized primers and a suitable kit for amplicon sequencing, a small number of representative strains was tested to screen MNP loci that could be detected in all strains to form the final MNP marker set and primer pools of MGV-Seq.

**FIGURE 1:**
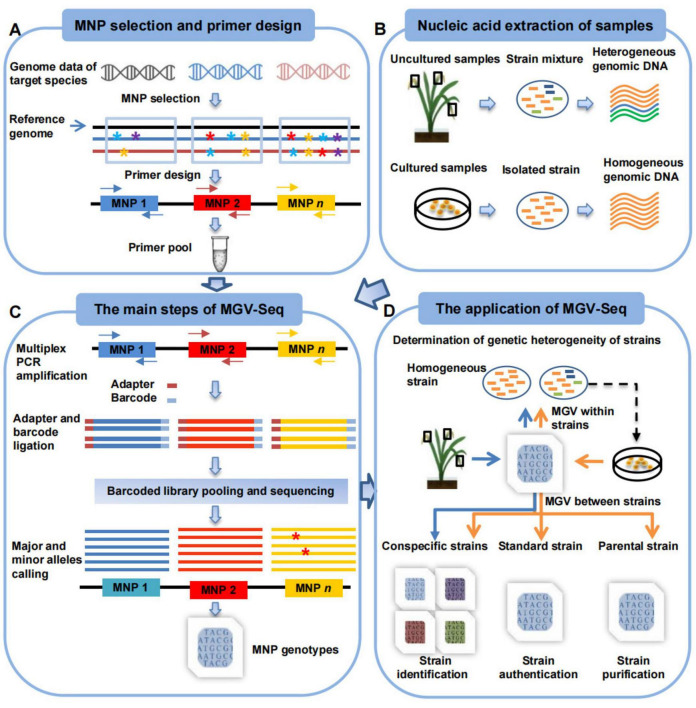
Workflow of MGV-Seq. **(A)** The design of MNP markers includes screening polymorphic candidate MNP loci from multiple genomes of the target microorganism, designing multiplex PCR primers for these loci, and validating and determining the valid MNP loci. * with different colors represents A, T, C, and G bases. Arrows of the same color in different directions represent a pair of primers. **(B)** Samples that MGV-Seq can detect. **(C)** The main steps of MGV-Seq include library preparation, high-throughput sequencing of the libraries, and bioinformatics analysis of the sequencing data. The red * represent the SNP variations among reads. **(D)** MGV-Seq-based procedure for heterogeneous microbial strain determination, strain authentication, purification monitoring, and identification. The arrows in the figure of two different colors represent two application procedures of MGV-Seq. The orange arrows correspond to homogeneous sample processing, while the blue arrows represent the experimental processes for heterogeneous samples. MNP, multiple dispersed nucleotide polymorphism; PCR, polymerase chain reaction; MGV, microbial genetic variation.

Next, MGV-Seq can be used to detect MNP markers in samples from pure culture to uncultured diseased host samples (Figures 1A,B). The PCR amplification products of each sample were ligated with a unique DNA barcode. Finally, the barcoded PCR products were mixed to form a library for high-throughput multiplex sequencing (Figure 1C). A customized computational tool was developed for MNP genotyping (Figure 1C). Briefly, we first assigned sequencing reads to samples based on DNA barcodes and mapped the reads to the reference genome. Next, all reads of an MNP locus were tallied to call for candidate alleles. The candidate allele supported by >50% of the total reads on a locus was defined as the major allele of the locus. All candidate minor alleles for an MNP locus were assumed to originate from the major allele due to amplicon sequencing errors. The true minor alleles of the locus were determined by strand bias filtering and statistical modeling (Figure 1C, see Materials and methods section). Finally, the MNP genotypes of each sample were constructed by integrating the alleles of all detected MNP loci.

A procedure for applying MGV-Seq to homogeneous and heterogeneous strain determination, strain identification, strain authentication, and monitoring strain microevolution was proposed (Figure 1D). The heterogeneity of the tested samples was determined based on the number of minor alleles detected in the MNP genotypes. By comparing the MNP genotypes of the tested sample with those of the conspecific, parental, or standard strains, MGV-Seq can be further used for identifying the dominant strain in the tested sample, monitoring the microevolution of daughter strain lines, and authenticating the strain to be used (see Materials and methods section).

#### 3.1.2 Reproducibility, accuracy, sensitivity, and specificity of MGV-Seq

This study used the phytopathogen *Xanthomonas oryzae* (*X. oryzae*) as a model microorganism. A set of 213 MNP markers was developed (Supplementary Table S1; see Materials and methods section) based on 458 genome assemblies of *X. oryzae* strains downloaded from the National Center for Biotechnology Information (NCBI), including 427 *Xoo* and 19 *Xoc* genomes (Supplementary Table S2). Widely used P1–P10 *Xoo* strains from IRRI (Quibod et al., 2016) and daughter lines from three different laboratories in Beijing, Wuhan, and Hainan, China, were collected (Supplementary Figure S1). The P1–P10 strains were short-handed for PXO61, PXO86, PXO79, PXO71, PXO112, PXO99, PXO145, PXO280, PXO339, and PXO341 in this study. Biological samples with the same name but from different laboratories are referred to as homonyms in this study, while DNA samples sequenced at the same run are referred to as a sequencing batch. Repeats of the same DNA tested in the same and different sequencing batches are called technical and batch repeats, respectively.

**FIGURE 2:**
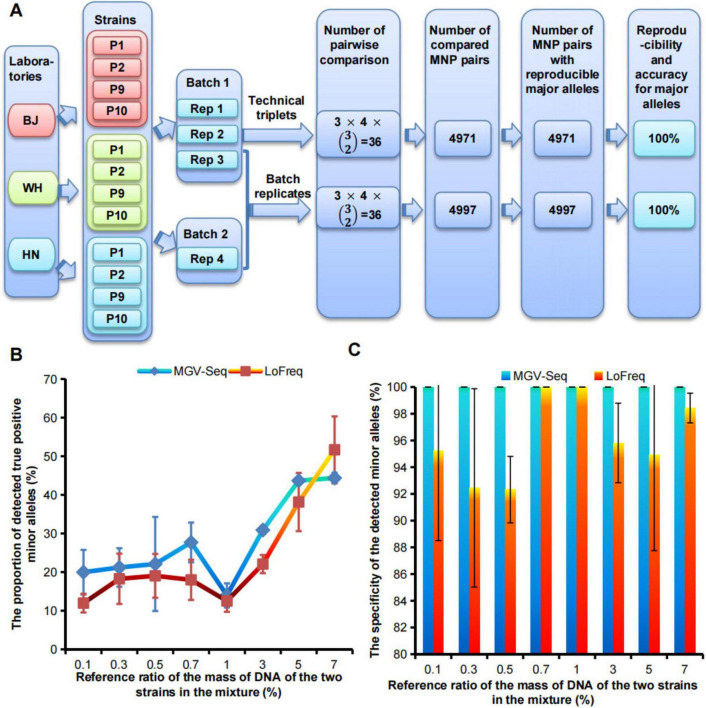
Evaluation of reproducibility, accuracy, sensitivity, and specificity of MGV-Seq. **(A)** The reproducibility and accuracy of MGV-Seq for detecting major alleles. **(B)** Sensitivity and **(C)** specificity of MGV-Seq for detecting low-abundance variant from artificial DNA mixtures of WH-P8 and WH-P6. BJ, Beijing; WH, Wuhan; HN, Hainan; Rep, replicate; MNP, multiple dispersed nucleotide polymorphism.

The dominant strain within each sample was identified by comparing the major alleles on the detected MNP loci with the reference genotypes of the conspecific strains. Therefore, the reproducibility of MGV-Seq for strain identification can be estimated using the reproducibility of MGV-Seq for detecting major alleles. A total of 4,971 MNP pairs among the 36 technical repeats and 4,997 MNP pairs between the 36 batch repeats were generated. The major alleles in all MNP pairs were 100% reproducible (Figure 2A and Supplementary Tables S3, S4). Reproducible major alleles were regarded as correct because there is only a tiny chance that major alleles of one MNP locus, detected by two repeats, were consistent but incorrect. Therefore, MGV-Seq demonstrated 100% reproducibility and accuracy for the technical and batch repeats for major allele detection. These 12 strains included three homonyms of the four strains. Pairwise comparison of homonymous strains was performed to examine the reproducibility of MGV-Seq for detecting differences across strain homonyms. A total $4\;\times \;\left(\begin{array}{c} 3\\ 2 \end{array}\right)\;=\;12$ pairs of homonymous strains were formed and each pair performed 4×4 = 16 repeats from the four replicates of each strain across the two batches. The reproducibility of MGV-Seq for detecting differences across strain homonyms was 100% (Supplementary Table S5).

The sensitivity and specificity of MGV-Seq were estimated based on its performance in detecting mixed samples. A mixed sample was characterized by the presence of minor alleles belonging to MNP genotypes of the different strains. MGV-Seq was tested on 14 single clones of WH-P8 designated as CP8-1–CP8-14, eight single clones of WH-P6, designated as CP6-1–CP6-8, and a series of mixtures by spiking the DNA of one WH-P8 clone into one WH-P6 clone at increasing reference ratios of 0.1, 0.3, 0.5, 0.7, 1, 3, 5, and 7% w/w, at the first sequencing batch (Supplementary Table S3). The genotyping results of the single clones and mixtures were analyzed and compared using the MGV-Seq-adapted computational pipeline developed by us and LoFreq, a previously reported ultra-sensitive SNP-based variation detecting tool (Wilm et al., 2012). Minor alleles detected in the mixture belonging to either WH-P6 minor alleles or WH-P8 major alleles were called true positive minor alleles (TP), while others were called false positive minor alleles (FP). The sensitivity and specificity of MGV-Seq were referred to as the lowest ratio of spiking DNA in the detected mixture and the percentage of TP to the sum of TP and FP, respectively. MGV-Seq reported 20–44.4% TP for 0.1–7% reference ratios of mixtures and achieved a perfect (100%) specificity (TP/(TP+FP)) at all mixture ratios (Figures 2B,C). In comparison, the percentages of TP detected by LoFreq for the 0.1–7% reference ratios ranged from 12–51.7% (Figure 2B). The results showed that the sensitivity of MGV-Seq and LoFreq in the detection of mixed samples was as low as 0.1%; however, LoFreq had lower specificity for six of the eight reference ratios tested, for example, 92.3% specificity at 0.5% mixture ratio (Figure 2C).

#### 3.1.3 WGS versus MGV-Seq

For technical comparison, whole genomes of the 10 *Xoo* strains from IRRI were sequenced (Supplementary Table S6). A total of 1,400 MNP loci detected by MGV-Seq in the 10 *Xoo* strains (IRRI-P1-IRRI-P10 at the second sequencing batch) were covered by the WGS data, and the major alleles were 100% verified by the WGS data (Supplementary Table S4), suggesting the genotyping reliability of MGV-Seq.

### 3.2 Applications of MGV-Seq

#### 3.2.1 Homogeneity and heterogeneity of the laboratory-adapted strains

**FIGURE 3:**
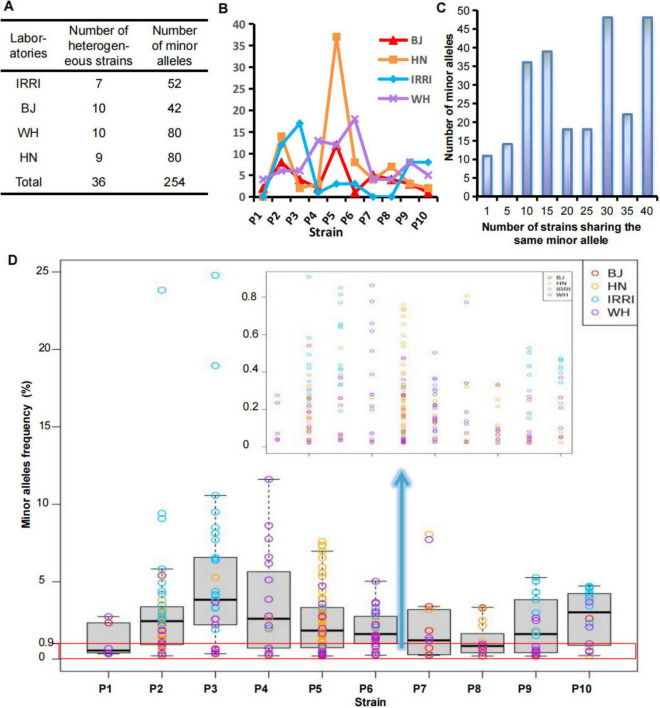
Determination of genetic homogeneity and heterogeneity of the laboratory-adapted strains. **(A,B)** Number of minor alleles of the *Xoo* strains from different laboratories. **(C)** Frequency of minor alleles of the *Xoo* strains from different laboratories. **(D)** Number distribution of strains sharing the same minor alleles. BJ, Beijing; WH, Wuhan; HN, Hainan; IRRI, International Rice Research Institute; *Xoo*, *Xanthomonas oryzae* pv. *oryzae*.

Genetic homogeneity or heterogeneity of the strain was determined based on the minor alleles detected at the MNP loci of each strain. We applied MGV-Seq to detect 40 laboratory-adapted *Xoo* strains corresponding to 10 distinct parental strains each with four homonyms at the second sequencing batch. The four homonymous strains were P1–P10 from the IRRI and daughter lines preserved in the Beijing, Wuhan, and Hainan laboratories, designated as IRRI-P1–IRRI-P10, BJ-P1–BJ-P10, WH-P1–WH-P10, and HN-P1–HN-P10, respectively (Supplementary Figure S1). On average, 1,676,528 reads mapped to 203 MNP loci per strain, with an average coverage of 8,245 folds per marker (Supplementary Table S3). No minor alleles were detected at the MNP loci in four of the 40 *Xoo* strains, which were considered homogeneous strains. MGV-Seq identified 254 minor alleles in the remaining 36 *Xoo* strains (Figure 3A), and the number of minor alleles in each strain ranged from 1–37 (Figure 3B), indicating that 90% of the tested laboratory-adapted strains were heterogeneous. Moreover, 31.9% (81 of 254) of minor alleles had a frequency <0.9% (Figure 3D; Supplementary Table S7), which was considered to be the lower limit of the SNP genotyping error rates (Bayer et al., 2017, Fang et al., 2017, Li, 2016, Sasaki et al., 2018). Therefore, accurately capturing low-frequency minor alleles using SNP genotyping-based methods is difficult.

#### 3.2.2 Identification of laboratory-cultured strains

**FIGURE 4:**
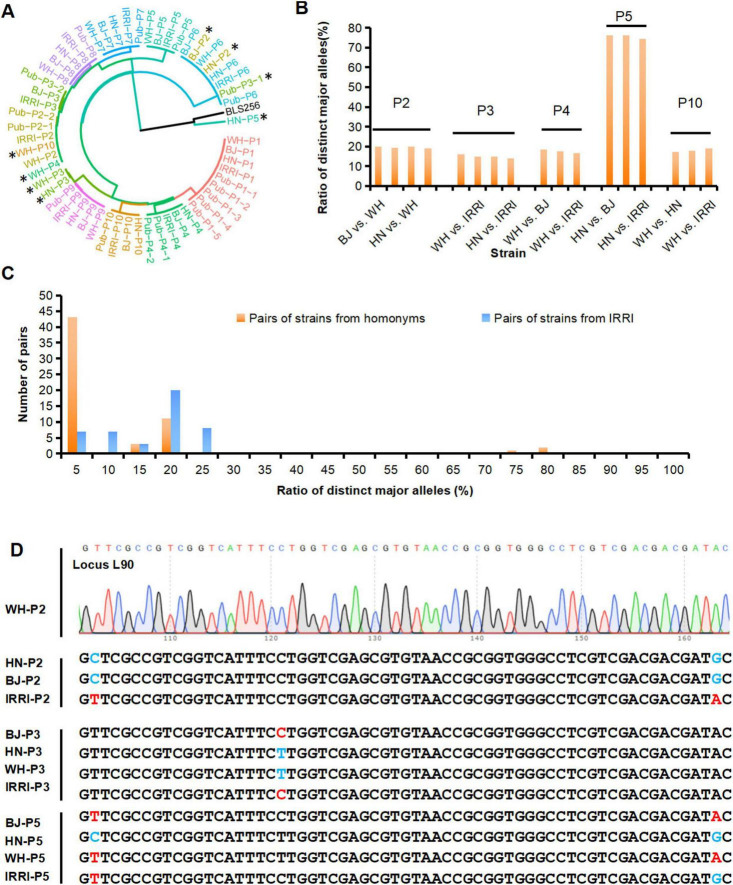
Identification and detection of genetic difference of homonymous strains from different laboratories. **(A)** MNP genotypes-based cluster analysis of the *Xoo* strains identified in this study and strains with published genomes (Pub-P1–Pub-P10). The published genomes, i.e., the four genomes of the P1 strains, were named Pub-P1-1, Pub-P1-2, Pub-P1-3, and Pub-P1-4. *represents an unexpected clustering of the genome, and the genome of BLS256 as the representative strain of *Xoc*. **(B)** The ratio of distinct major alleles between the *Xoo* homonyms derived from different laboratories. **(C)** Distribution of the ratios of distinct major alleles of strains homonyms and the IRRI strains. **(D)** Validation of genotypes of MNP loci among homonymous strains. MNP, multiple dispersed nucleotide polymorphism; *Xoo*, *Xanthomonas oryzae* pv. *oryzae*. BJ, Beijing; WH, Wuhan; HN, Hainan; IRRI, International Rice Research Institute.

We then identified these 40 strains by conducting a genetic cluster analysis based on the MNP genotypes of the 40 strains, the previously published genomes of P1–P10, named Pub-P1–Pub-P10 (Quibod et al., 2016, Salzberg et al., 2008, Song et al., 2023). and the genome of BLS254, representative strain of *Xoc* (Feng et al., 2015, Hajri et al., 2012, Lee et al., 2005, Quibod et al., 2016, Salzberg et al., 2008). As illustrated by the cluster dendrogram, 39 of the 40 strains were *Xoo* pathovars, but one (HN-P5) was distant from all analyzed *Xoo* strains but closer with *Xoc* strains, which was identified as *Xoc* (Figure 4A). Alarmingly, BJ-P2 and HN-P2 were not clustered with their parental strains but were closest to the P6 strains, WH-P3 and HN-P3 were closest to the P9 strains, WH-P4 and WH-P10 were closest to some P2 strains, and HN-P5 were not clustered with any *Xoo* strains, respectively, indicating that there was a deviation between the identified and claimed identities of these strains. Pairwise comparison of P1–P10 homonymous strains from the four laboratories was performed to examine MGV across strain homonyms. Of the total $10\;\times \;\left(\begin{array}{c} 4\\ 2 \end{array}\right)\;=\;60$ pairs, 17 (28.3%) had distinct major alleles with ratios of 14.0–76.2% (Figure 4B; Supplementary Table S8), suggesting that MGV was prevalent among these strain homonyms. An ultra-high ratio of major allele differences existed between HN-P5 and other P5 strains, confirming the MGV-Seq genotyping result that HN-P5 belonged to *Xoc* rather than *Xoo*.

The significant degree of genetic discrepancy in the reference strain across daughter lines raised concerns about the origin of the genetic differences. Thus, we analyzed the distribution of distinct major alleles among the daughter lines and different strains. Different *Xoo* strains diverged for much longer than daughter lines and thus should have accumulated more mutations. Distinct major alleles among the daughter lines (14.0–76.2%, Supplementary Table S8) were greater than those among different strains (0–23.5%, Supplementary Table S9), and the distribution of distinct major alleles in the daughter lines was discontinuous (Figure 4C). We further analyzed the distribution of minor alleles among the 40 strains. Eleven (4.3%) of the 254 minor alleles appeared only once and differed from the major alleles at any locus, suggesting that spontaneous mutations may have caused them. The remaining 243 (95.7%) minor alleles were the same as the major alleles of the other 1–39 *Xoo* strains tested together (Figure 3C; Supplementary Table S7). These results strongly suggested that human error, such as sample mislabeling or contamination, rather than spontaneous mutations, might be the primary factor underlying the genetic discrepancy in the examined *Xoo* daughter lines captured by MGV-Seq. Genotypes of a randomly selected MNP locus in the strain homonyms were successfully validated using Sanger sequencing (Figure 4D).

**FIGURE 5:**
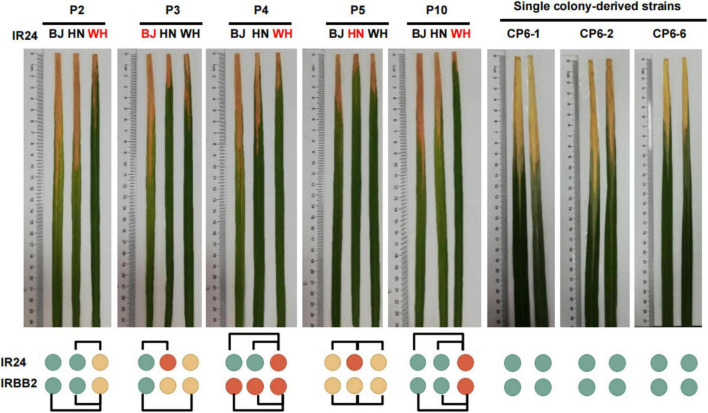
Pathogenicity differences among the *Xanthomonas oryzae* pv. *oryzae* strain homonyms. Names marked red indicates the strain with major alleles distinct from its homonyms. Green, yellow, and red circles represent disease reaction levels of susceptible, moderately resistant, and resistant, respectively. CP6-1, CP6-2, and CP6-6 are single colony-derived strains from WH-P6. BJ, Beijing; WH, Wuhan; HN, Hainan. Two-tailed Student’s *t*-tests were performed to compare lesion lengths between homonymous strains. Homonymous strains with a *p*< 0.005 were connected with black frame.

A large number of MGV of *Xoo* homonyms suggested that these strains might have distinct pathogenicity, one of the most important phenotypic features of *Xoo*. We investigated the pathogenicity of P2, P3, P4, P5, and P10 homonyms from Wuhan, Beijing, and Hainan on the susceptible rice variety IR24 and a near-isogenic line IRBB2 carrying the *Xoo* resistance gene *Xa2* (Figure 5; Supplementary Table S10). Most of the 10 pairs of strain homonyms with distinct major alleles induced significantly different disease reactions in IR24 and IRBB2 rice varieties, showing the effect of MGV on pathogenicity reproducibility. One (WH-P3 vs. HN-P3) out of the five pairs of *Xoo* homonyms without distinct major alleles had a difference in lesion lengths (LL) of leaves, which may be due to the effect of minor alleles on pathogenicity reproducibility.

#### 3.2.3 Identification of Xanthomas oryzae strains detected in rice leaf samples

**FIGURE 6:**
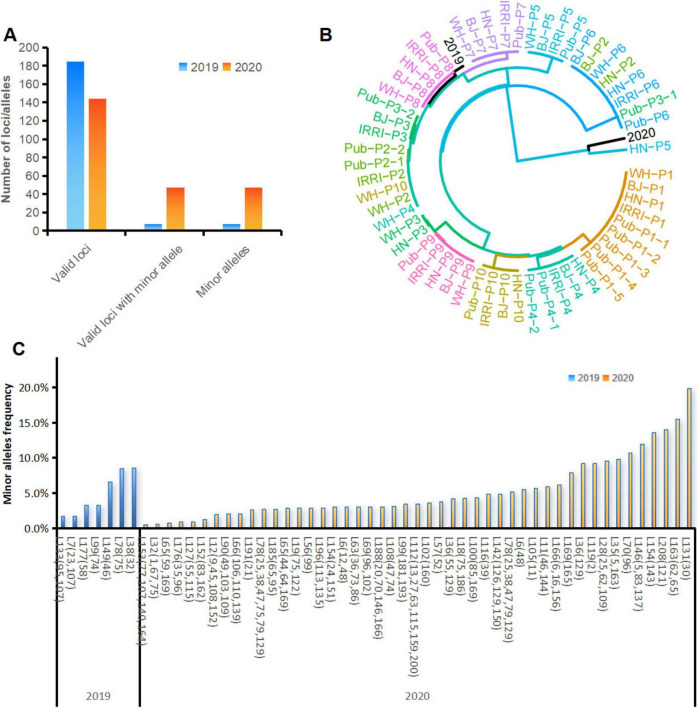
Strain identification and variation detection in rice leaf samples. **(A)** Number of valid MNP loci, valid MNP loci with minor alleles and number of minor alleles detected in the DNA of rice leaf samples collected in 2019 and 2020. **(B)** Cluster analysis of the strains identified in rice leaf samples with the *Xanthomonas oryzae* pv. *oryzae* strains used in this study. **(C)** Positions and frequencies of the minor alleles detected in the DNA of the rice leaf samples collected in 2019 and 2020. The numbers in parentheses represent the base positions of the minor alleles within the MNP loci. MNP, multiple dispersed nucleotide polymorphism; BJ, Beijing; WH, Wuhan; HN, Hainan.

We used MGV-Seq to detect *X. oryzae* strains in rice leaves—with similar leaf blight symptoms—collected from the same paddy field in 2019 and 2020. MGV-Seq reported 185 and 144 valid MNP loci in the two rice leaf samples, respectively. And 7 of the 185 valid MNP loci and 47 of the 144 valid MNP loci had minor alleles (Figure 6A). The number of minor alleles of each MNP loci was one, with frequencies ranging from 0.5 to 19.8% (Figures 6B,C; Supplementary Table S11), suggesting the presence of low-frequency genetic variation in these samples. We identified the strains in the two rice leaf samples by comparing the major alleles of MNP loci detected with the 458 genomes in the reference database of *X. oryzae*. To illustrate the genetic distance between the rice sample genotypes and the reference strains, we plotted a clustering tree (Figure 6B). For the clarity and readability of the clustering tree, the 10 strains, with genomes closest to that of P1-P10, in the *X. oryzae* reference database based on the calculated average nucleotide identity, and the X-representative strains were selected as the reference strains for plotting the clustering tree. All the results presented in the heatmap (Supplementary Figure S3) and the clustering tree showed that the dominant strain in the 2019 sample was closest to the PXO211 strain of *Xoo*, whereas that in the 2020 sample was closest to the L8 strain of *Xoc* (Figure 6B; Supplementary Table S12).

#### 3.2.4 Authentication of strains

The object of strain authentication is to determine whether the homonymous strains used at different periods (from the same laboratory or among different laboratories) are genetically consistent with their standard strain. The feasibility of MGV-Seq-based strain authentication was validated via the authentication of P6 and P8 homonymous lines. We took IRRI-P6 and IRRI-P8 as the standard strains, respectively. The homonymous lines of P6 included BJ-P6, HN-P6, WH-P6 and eight WH-P6 clones (CP6-1 – CP6-8) and that of P8 included BJ-P8, HN-P8, WH-P8 and 14 WH-P8 clones (CP8-1 – CP8-14). A pairwise comparison of the MNP major alleles of WH-P6, WH-P8 and their single-colony strains with IRRI-P6 and IRRI-P8, respectively, revealed that all the daughter lines showed 100% major allele similarity (MAS) with their standard strains IRRI-P6 and IRRI-P8, demonstrating that they were authenticated properly (Supplementary Table S13). Indeed, pathogenicity identification showed no significant differences among the three homogeneous WH-P6 clones (Figure 5; Supplementary Table S10).

Overall, these results demonstrated that MGV-Seq can identify microorganisms at the strain level. Additionally, it can accurately detect low-abundance variations in cultured strains and uncultured host-contaminated samples (directly). Thus, MGV-Seq was readily available for daughter-line microevolution monitoring and strain authentication.

## 4 Discussion

In this study, we focused on the development, performance evaluation, and demonstration of diverse applications of the MGV-Seq method. MGV-Seq genotyped a high-density panel of MNP markers within the genomes of target species using target enrichment and ultra-deep sequencing. Our results revealed 100% reproducibility and 100% accuracy for MGV-Seq in detecting major alleles and a comparable sensitivity but superior specificity in detecting genetic variations compared to the known exceptionally sensitive variant detection tool (Wilm et al., 2012; Figure 2).

The prominent features of MGV-Seq can be attributed to several factors, as follows: First, MGV-Seq introduced as many markers as possible to minimize or avoid profiling errors; for example, MGV-Seq screened 213 MNP markers of the 4.49 Mbp genome of *Xoo*, forming a high-density panel of markers for a relatively small microbial genome (Supplementary Table S2). Second, in our study, MGV-Seq easily achieved ultra-high sequencing coverage—8,425 folds per MNP marker (Supplementary Table S3). Such a dense panel of markers and such deep sequence coverage on the MNP markers would be ideal for genetic studies to accurately and sensitively detect and identify strains and analyze many low-frequency genetic variations with minimal profiling errors.

The prominent features of MGV-Seq make it markedly advantageous over existing methods for a wide range of applications, as illuminated in this study: (1) *Strain identification*: MGV-Seq achieved strain-level identification for pure culture and disentangled the genetic diversity of target microorganisms in uncultured diseased host samples, all with very high throughput and resolution. MGV-Seq overcomes the limitations of the electrophoresis band-based method commonly used for identifying cultured samples in terms of resolution, accuracy, and dependence on standard strains. It also shows advantages over the metagenomics-based method commonly used for identifying uncultured host samples regarding data volume, cost, analysis, and resolution. **(2)** *Monitoring strain microevolution*: MGV-Seq can accurately and sensitively capture low-frequency minor alleles that are difficult to capture using SNP genotyping-based methods (Figure 3D; Supplementary Table S9). This characteristic confers MGV-Seq an advantage in detecting variation of strains at early periods, which is important for monitoring strain microevolution and new variants. However, it should be noted that phylogenetic discovery bias occurs when the molecular markers are derived from biased taxonomic sampling markers (Pearson et al., 2004). As shown in this study, the cluster dendrograms of IRRI-P1–IRRI-P10 based on variations detected via MGV-Seq and WGS were completely consistent, suggesting that cluster analysis with multi-polymorphic markers is beneficial in avoiding the occurrence of this preference. (3) *Monitoring strain purification*: A pure culture is indispensable for infectious disease research. In this study, to demonstrate the feasibility of MGV-Seq-monitored strain purification, one round of single-colony isolation of WH-P6 and WH-P8, which MGV-Seq detected at 18 and 4 minor alleles (Supplementary Table S3), respectively, was performed using the spread plate and streak plate methods. All 22 clones showed 100% MAS with the parental strain, and the 15 clones harbored no minor alleles (Supplementary Table S3), all of which were homogeneous strains. This result showed that the clones were properly purified and that MGV-Seq could effectively monitor the strain purification process of heterogeneous samples. 4) Strain authentication. Heterogeneity of homonymous strains has been reported in several human pathogenic microbial species, such as *Pseudomonas aeruginosa* from 10 laboratories (Chandler et al., 2019) and the 13 daughter lines of the tuberculosis vaccine *Mycobacterium bovis* BCG (Garcia Pelayo et al., 2009). Our study reported genotype and phenotype discrepancies across the homonymous strains of *X. oryzae*. One solution to this serious problem is to introduce a mechanism of microorganism authentication, similar to that in human cell lines, to ensure that the same experimental materials are used in multiple experiments and across different laboratories. Therefore, the key index of technology for strain authentication was the high reproducibility and comparability of the genotyping results between experimental batches, ensuring result reproducibility and comparability across different laboratories. For the relatively small genome size of bacteria, compared with human cell lines, researchers may prefer the WGS method for strain authentication. However, in cases with large sample sizes, such as a study by (Song et al., 2023) which had more than 240 *Xoo* strains, or with large-genome microorganisms (fungi), the WGS method is still expensive (Guo et al., 2019). We have demonstrated that MGV-Seq showed 100% technical and batch reproducibility in detecting MNP major alleles (Figure 2) and achieved a resolution approaching that of WGS-based methods. Using the barcodes and chips on existing platforms, for example, 768 barcodes and 550 Ion chips in the Ion Torrent sequencing platform, up to 1536 samples can be tested in a single sequencing run, enabling cost-effective MGV-Seq of large sample sets. The prominent features of MGV-Seq make it readily applicable to strain authentication (Table 1; Supplementary Table S13). Furthermore, because the heterogeneity of homonymous strains has also been observed in human pathogens, we strongly suggest MGV-Seq authentication of biological samples from other microorganisms.

**TABLE 1:** Major allele similarity between single colony-derived strains and their parental strains.

Single colony-derived strains	Parental strains	Number of compared loci	Number of compared loci with same major alleles	Major allele similarity
CP6-1	WH-P6	139	139	100%
CP6-2	WH-P6	108	108	100%
CP6-3	WH-P6	135	135	100%
CP6-4	WH-P6	136	136	100%
CP6-5	WH-P6	140	140	100%
CP6-6	WH-P6	126	126	100%
CP6-7	WH-P6	129	129	100%
CP6-8	WH-P6	134	134	100%
CP8-1	WH-P8	172	172	100%
CP8-2	WH-P8	181	181	100%
CP8-3	WH-P8	182	182	100%
CP8-4	WH-P8	178	178	100%
CP8-5	WH-P8	135	135	100%
CP8-6	WH-P8	168	168	100%
CP8-7	WH-P8	169	169	100%
CP8-8	WH-P8	168	168	100%
CP8-9	WH-P8	166	166	100%
CP8-10	WH-P8	166	166	100%
CP8-11	WH-P8	165	165	100%
CP8-12	WH-P8	166	166	100%
CP8-13	WH-P8	142	142	100%
CP8-14	WH-P8	154	154	100%

CP6-1∼CP6-8 and CP8-1∼CP8-14: single colony-derived strains.

In addition to the applications that have been illuminated in this study, MGV-Seq is potentially applicable to high-throughput and low-cost detection of medical and forensic samples—which typically have high host contamination or low biomass—and for monitoring low-frequency genetic variations. Our study demonstrated that MGV-Seq could sensitively detect low-abundance variations directly from uncultured samples (Figure 6). The targets of multiplex PCR can be in the thousands (Li et al., 2017). Thus, MGV-Seq is potentially feasible for detecting and typing multiple pathogen species in one panel in clinical samples, such as respiratory and intestinal tract samples, and plant quarantine samples, such as imported seeds and seedlings, by distributing the MNP loci to multiple pathogen species.

More importantly, the main steps for MGV-Seq are simple and compatible between sequencing platforms. Though MGV-Seq is highly dependent on the Ion Torrent technology in this study, MGV-Seq was readily available to any sequencing platform by adjusting the adaptors to the sequencing platforms on primers. In our previous study on plant variety identification, the Illumina high-throughput sequencing platform was used (Fang et al., 2021). Besides, we have already developed MNP panels for detecting SARS-CoV-2 based on Illumina and Beijing Genomics Institute sequencing platforms (data not shown). Sequencing data from any sequencing platform and laboratory can be shared through public databases. The genotypes can be derived and compared by our provided containerized tools, which can be compared freely and efficiently across laboratories.

Notably, several technical challenges should be noted. Firstly, it is well known that different sequencing platforms harbor their own sequencing bias (Quail et al., 2012, Suzuki et al., 2011), potentially leading to discordant genotypes for one sample. Therefore, it is necessary to consider comparing genotypes from different sequencing platforms. Furthermore, the ultra-sensitivity of MGV-Seq has made it sensitive to aerosol contamination and unavoidable technical problems of amplicon sequencing, such as PCR and sequencing errors, index hopping, and demultiplexing errors, which could lead to the generation of incorrect alleles. These alleles are often low-frequency and are confused with true low-frequency alleles. To identify the true low-frequency alleles, an optimal statistical analyzing pipeline (strand bias filter and statistical model) was adopted to remove incorrect alleles (see Materials and methods section). The high specificity of the minor alleles detected in the artificial DNA mixtures of the P6 and P8 strains proved the reliability of our statistical analysis pipeline for low-frequency minor allele detection.

In summary, the multifaceted capabilities of MGV-Seq underscore its potential to revolutionize microbial genomics across diverse fields. It integrates high - resolution strain - level discrimination, sensitivity to low - frequency variants, and cost - effectiveness. Its adaptability to multiple sequencing platforms enhance global standardization of strain authentication and comparative genomics. Beyond the demonstrated robust detection of pathogens in field samples, MGV-Seq’s capacity to detect pathogen diversity in complex matrices (e.g., clinical samples with host contamination, low-biomass forensic specimens) positions it as a versatile tool for precision medicine, public health surveillance, and biosecurity. Crucially, MGV-Seq’s emphasis on reproducibility and cross-laboratory comparability highlights the need for standardized microbial authentication, akin to human cell line authentication systems. Its framework for tracking genetic variation at early stages of evolution could also inform strategies against antimicrobial resistance or pathogen adaptation. In the future, as a foundational technology for microbiology, MGV-Seq is poised to fostering global collaboration through shared databases and containerized tools, seamless adaptation to new organisms and emerging challenges.

## Data Availability

The data supporting this study’s findings are openly available in the NCBI BioProject database (https://www.ncbi.nlm.nih.gov/bioproject/) at accession number PRJNA679673. In addition, the codes generated during this study are available at: https://github.com/SystemsBiologyOfJianghanUniversity/MGV-Seq.

## Footnotes

1
https://ampliseq.com/

2
http://hannonlab.cshl.edu/fastx_toolkit/index.html

## Supplementary material

Supplementary Figure S1 The transmission time and route of the *Xanthomonas oryzae* pv. *oryzae* strains used in this study. The strains enclosed in the black box were used in this study.

Supplementary Figure S2 The customized computational pipeline for MNP genotyping includes calling candidate alleles, identifying major alleles, and detecting true minor alleles. MNP, multiple dispersed nucleotide polymorphism; SNPs, single nucleotide polymorphisms.

Supplementary Figure S3 The heatmap of pairwise comparisons of homonymous strains.
