# Supplementary figures and images for: MGV-seq: a sensitive and culture-independent method for detecting microbial genetic variation
Source: Front Microbiol. 2025 Jun 25;16:1603255. doi: 10.3389/fmicb.2025.1603255 (PMC12237947; doi:10.3389/fmicb.2025.1603255)

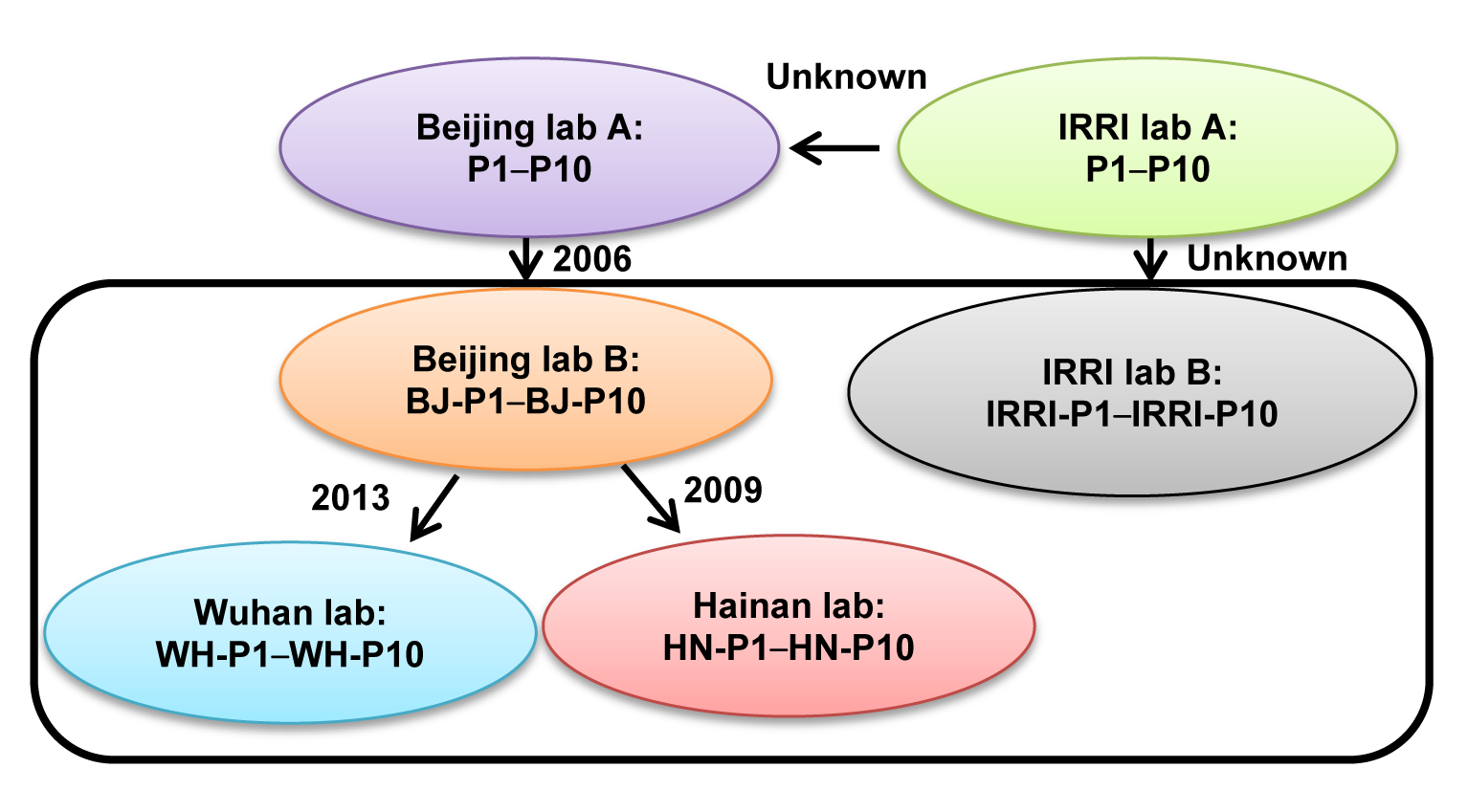

Supplement: Supplementary Figure S1 — The transmission time and route of the Xanthomonas oryzae pv. oryzae strains used in this study. The strains enclosed in the black box were used in this study. [file Image_1.TIF]

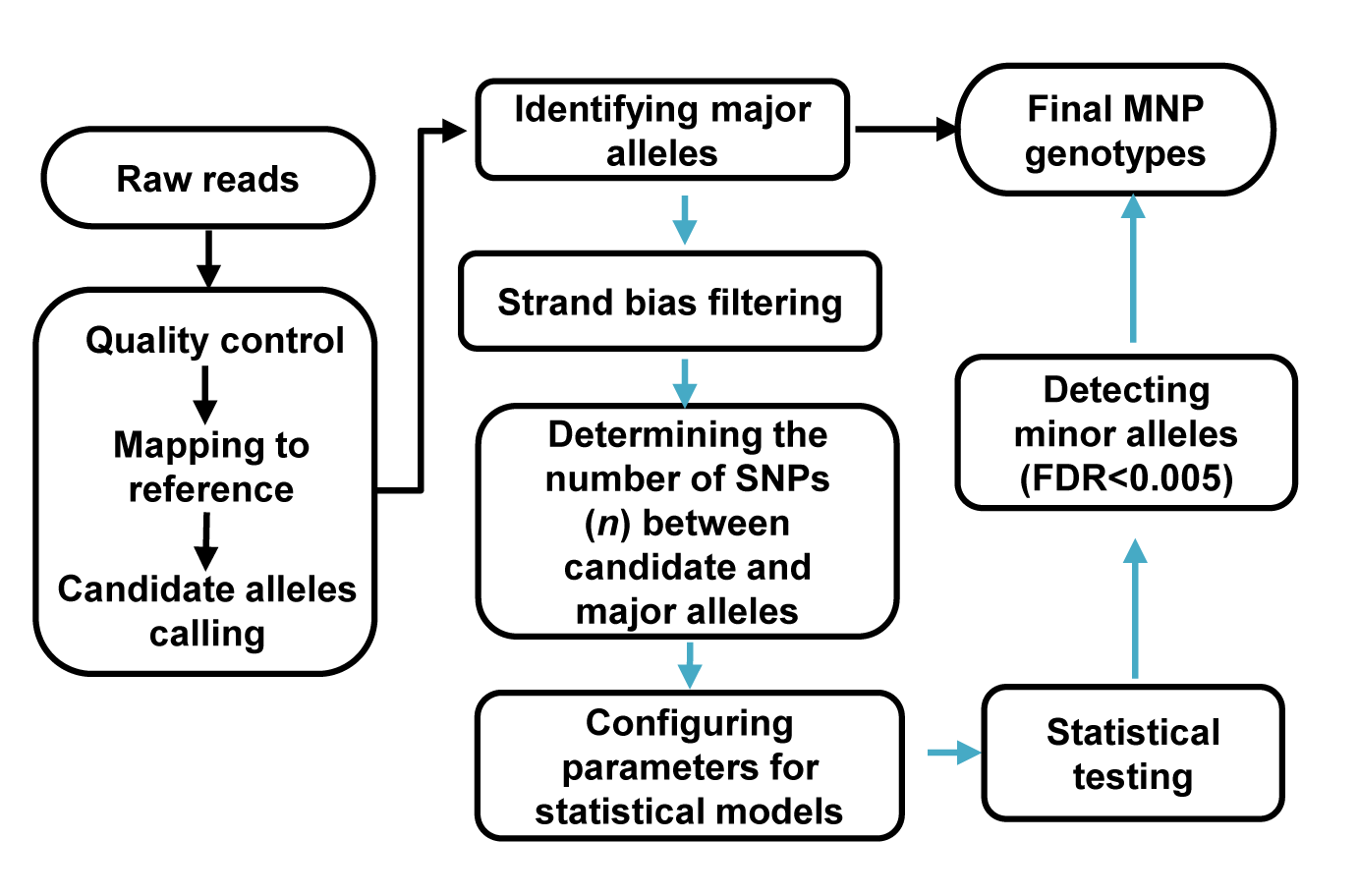

Supplement: Supplementary Figure S2 — The customized computational pipeline for MNP genotyping includes calling candidate alleles, identifying major alleles, and detecting true minor alleles. MNP, multiple dispersed nucleotide polymorphism; SNPs, single nucleotide polymorphisms. [file Image_2.TIF]

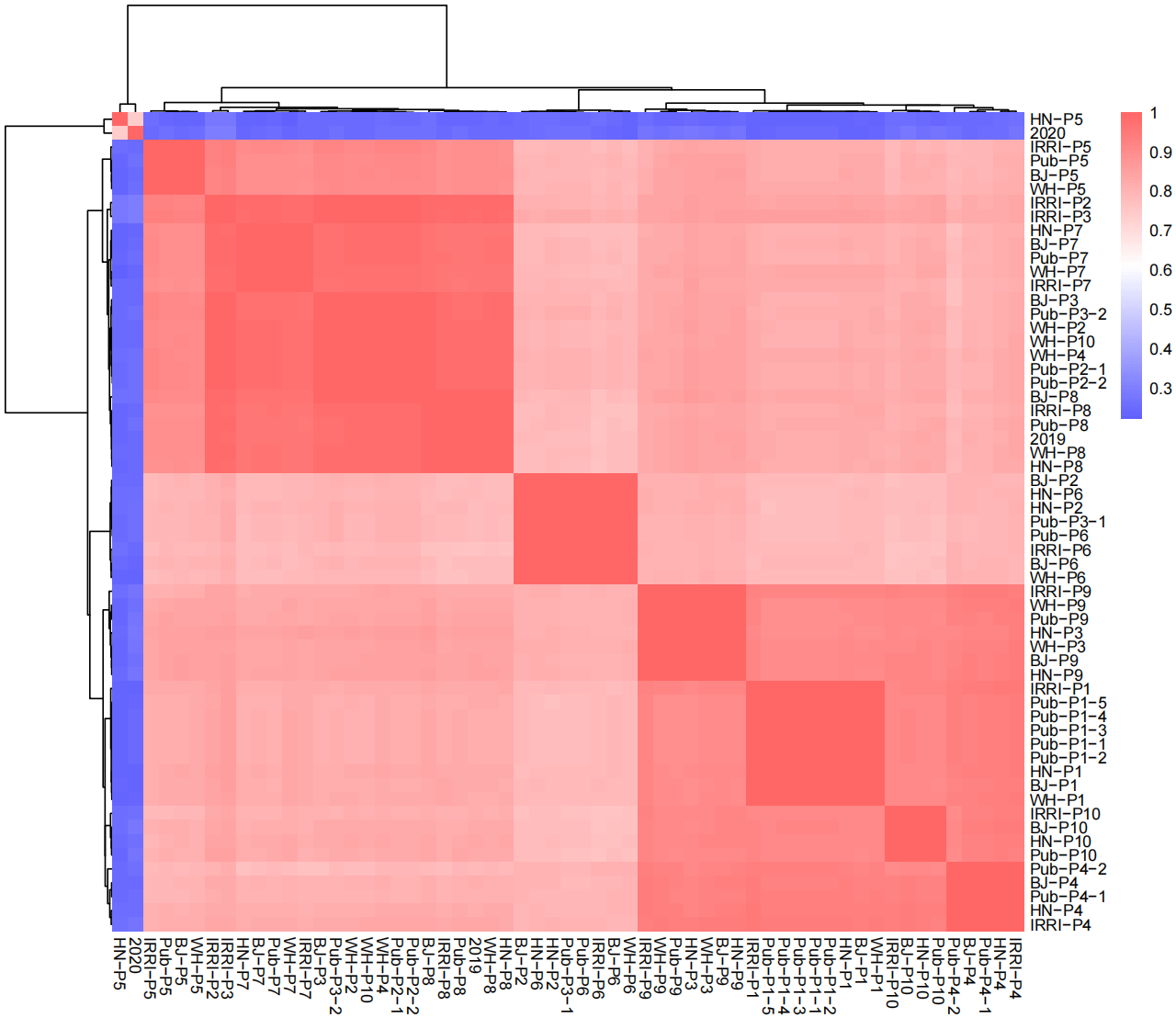

Supplement: Supplementary Figure S3 — The heatmap of pairwise comparisons of homonymous strains. [file Image_3.TIF]
